# Epsom College

**Published:** 1895-07-13

**Authors:** 


### The Institutional Workshop.

### EPSOM COLLEGE.

### Visit of T.R.H. the Prince and Princess of Wales.

Under Dr. Holman's able guidance Epsom College is rapidly becoming one of the most promising and popular of public schools. After years of hard work he has at length succeeded in arranging to place the buildings formerly occupied by the pensioners at the disposal of the college by providing out-pensions of sufficient amount to enable the residents to become non-resident pensioners, with advantage to themselves and the school. The enlargement of the school on a free and wide basis has long been desired. Last year the Council obtained a new Act of Parliament which enabled the college to be opened to all classes, whilst it will still afford special advantages to the sons of medical men. Mr. J. Lumsden Propert, one of the most popular men in the profession, and the son of the founder, good John Propert, must have been proud indeed when he realised the importance of the ceremony on Monday, when H.R.H. the Prince of Wales laid the foundation stone of a new building which is to provide accommodation for 100 of the younger boys, who will thus, in accordance with modern ideas, be separated from the main body of the school. The school has now for some years been quite full, and 250 boys are being educated within its walls. Its scholars have succeeded in entering with credit the medical as well as other professions, and they have distinguished themselves by winning many open scholarships at the Universities and at the London hospitals. Fifty boys, sons of medical men, are educated on the foundation free of cost, in addition to many others who receive assistance by means of scholarships and exhibitions. A great point is made of the science teaching, so as to meet the wants of boys preparing for a medical career, and the course of education seeks to impart to all classes of boys a healthy and liberal training, both mental and physical. Monday was a perfect day, so far as weather was concerned, and the presence of H.R.H. the Princess of Wales and the Princess Victoria added *éclat* to the proceedings. It was pleasant to notice the warm welcome which the large and distinguished assembly extended to H.R.H. the Princess, and to see how much it was appreciated by her. The Princess takes a deep interest in education, and her schools at Sandringham, which occupy much of her time and thought, are models of their kind.

The proceedings at the laying of the foundation-stone were commendably brief. The head master, the Rev. T. N. Hart-Smith, M.A., after the presentation of a bouquet of flowers to the Princess by Miss Alice Hart-Smith, his daughter, read an address. This address reminded the assembly that the Prince Consort originally intended to lay the first stone of the college more than forty years ago, and that on June 25th, 1855, he honoured the opening ceremony with his presence, being accompanied by the Prince of Wales on that occasion. The Queen has bestowed her royal patronage on this institution, and the address testified to the heartfelt appreciation of the interest always shown by H.R.H. the Prince of Wales, to which his presence, and that of her Royal Highness, at the ceremony on Monday bore eloquent testimony. After prayer by the Archdeacon of Middlesex, the first head master of the college, the Prince proceeded to lay the foundation-stone, with the assistance of Sir A. W. Blomfield, whose appointment as architect to the college may be taken as a guarantee that the new buildings will be as perfect as it is possible to make them. When the stone had been well and firmly laid, the Old Hundredth psalm was sung by the boys, and the Prince and Princess proceeded to the college library, where the house masters and staff were presented, together with the prefects of the school. Refreshments were provided on the lawn, and the 1,200 visitors evidently enjoyed the garden party thoroughly. We must not forget to mention the excellent music discoursed by the band of the Artists' Corps, which was admirably conducted by the bandmaster, who evidently felt with his confrères that the honour of this corps was in their hands, and they must exert themselves to the utmost to secure that the music should at any rate be of excellent quality. The East Surrey Volunteers supplied a guard of honour at the Epsom Downs Station, and the Cadet Corps of the College formed a guard of honour at the grand entrance on the arrival and departure of the Prince and Princess of Wales. We congratulate Dr. Holman and the council upon the excellent arrangements made for the comfort of the guests. We believe that this memorable ceremony is well calculated to popularise the college, to extend its usefulness, and to secure the subscription of sufficient funds to provide for the large outlay upon the new buildings, and to enable the Council to complete the other necessary works now in progress, including the enlargement of the school chapel.

### Speech of the Prince of Wales.

The Prince of Wales said: Ladies and gentlemen, I am very much obliged to Mr. Hart Smith for the address we have just heard, and I can assure you and all who take an interest in the welfare of Epsom College that it gives great pleasure to the Princess and myself to take part in this ceremony. The account you have given of the College shows that it is in a prosperous condition, and long may it continue so! I take a deep interest in the Institution, because forty years ago I was here with my father, who was not able on that occasion to lay the foundation-stone. I am glad to be able to do so myself, and to express the hope that the new building may increase in every sense the usefulness of the College.

